# *Drosophila TRPA* Channel *Painless* Inhibits Male–Male Courtship Behavior through Modulating Olfactory Sensation

**DOI:** 10.1371/journal.pone.0025890

**Published:** 2011-11-02

**Authors:** Kaiyu Wang, Yanmeng Guo, Fei Wang, Zuoren Wang

**Affiliations:** 1 State Key Laboratory of Neuroscience, Institute of Neuroscience, Shanghai Institutes for Biological Sciences, Chinese Academy of Sciences, Shanghai, China; 2 Graduate School of Chinese Academy of Sciences, Chinese Academy of Sciences, Bejing, China; AgroParisTech, France

## Abstract

The *Drosophila melanogaster TRPA* family member *painless*, expressed in a subset of multidendritic neurons embeding in the larval epidermis, is necessary for larval nociception of noxious heat or mechanical stimuli. However, the function of *painless* in adult flies remains largely unknown. Here we report that mutation of *painless* leads to a defect in male–male courtship behavior and alteration in olfaction sensitivity in adult flies. Specific downregulation of the expression of the Painless protein in the olfactory projection neurons (PNs) of the antennal lobes (ALs) resulted in a phenotype resembling that found in *painless* mutant flies, whereas overexpression of Painless in PNs of *painless* mutant males suppressed male–male courtship behavior. The downregulation of Painless exclusively during adulthood also resulted in male–male courtship behavior. In addition, mutation of the *painless* gene in flies caused changes in olfaction, suggesting a role for this gene in olfactory processing. These results indicate that functions of *painless* in the adult central nervous system of *Drosophila* include modulation of olfactory processing and inhibition of male–male courtship behavior.

## Introduction

Transient receptor potential (TRP) channels play important roles in a variety of sensory systems [1]. In mammals, *TRPA1* is critical for nociception and may contribute to cold sensation [2]–[4]. As a *Drosophila TRPA* channel [5], the *painless* gene was first identified as essential for the sensation of high temperature (above ∼39°C) and for noxious mechanical stimulation in the larvae [6], [7]. In addition, *painless* has been reported to be involved in sugar-stimulated neural excitation and avoidance in larvae [8], in wasabi sensation and noxious heat sensation in adult flies [9], [10], as well as in negative gravity sensation [11]. The expression pattern of the Gal4 enhancer-trap allele *painless-Gal4* (*pain^Gal4^*) suggests that *painless* may be endogenously expressed in the adult fly brain [9], [10], but likely functional consequences of this expression are largely unknown.

The *Drosophila* olfactory system is critical for its detection of volatile chemicals in the external environment [12]–[14]. These chemical cues are first detected and converted into electric signals by the primary olfactory receptor neurons (ORNs). After further processing by secondary projection neurons (PNs) and local interneurons (LNs) of the antennal lobes (ALs), this information is then conveyed to higher brain regions to direct appropriate behaviors, including foraging, fighting and courtship [15]. Pheromones act as important cues for gender identification in flies [16]. Thus, the *Drosophila* olfactory system, which detects volatile pheromone cues, plays an important role in courtship behavior. Dysfunction of the primary pheromone-sensing ORNs impairs pheromone detection and results in aberrant courtship behavior [17]. Additionally, activity of higher-order olfactory neurons, where information from ORNs is processed and modified, may also influence *Drosophila* courtship behavior.

In this study, we first examined the endogenous expression pattern of the *painless* gene in the adult *Drosophila* brain. Analysis of the *painless* mutant flies led to the observation that their olfactory sensitivity was decreased. In addition, we identified abnormal male-male courtship behavior. Manipulating of *painless* expression in specific brain regions revealed that its expression in the PNs of the ALs was critical for preventing the male–male courtship behavior. Further experiments using time-specific knockdown of *painless* under the regulation of the temperature-sensitive Gal80 protein (Gal80^ts^) showed that *painless* expression in the adult brain was essential for normal courtship behavior. Thus, *painless* may function in PNs to regulate male courtship behavior.

## Materials and Methods

### Drosophila Stocks and Culture

Flies were cultured on standard cornmeal–agar–molasses medium at 25°C with a relative humidity of 50–70%. The *painless^1^*, *painless-Gal4*, and *UAS-painless^AR9^* lines generated in the *w1118* background were a generous gift from Dr. D. Tracey (Duke University, Durham, NC, USA). The *CS* strain was used as a *WT* control. The *painless^3^* and *painless^4^* lines in a *w1118* background were obtained from the Szeged *Drosophila* Stock Center (Szeged, Hungary). The *GH146-Gal4*, *Poxn-Gal4-13-1*, *c507-Gal4*, and *UAS-shibire^ts^* lines were kindly provided by Drs. R.F. Stocker (University of Fribourg, Fribourg, Switzerland), M. Noll (University of Zurich, Zurich, Switzerland), D. Armstrong (University of Edinburgh, Edinburgh, UK), and T. Kitamoto (University of Iowa, Iowa City, USA), respectively. The *UAS-pain-RNAi* lineS were obtained from the Vienna *Drosophila* RNAi center (stock No. v39477 and v39478). Other strains were from the Bloomington *Drosophila* Stock Center (stock No. 854, 8769, 7018, 5130, and 23129). All flies used in behavior experiments have been back-crossed to *CS* flies for at least four generations.

### Behavioral Assays

All behavioral assays were carried out at 25±1°C with 40–60% relative humility at zeitgeber time ZT1-ZT8, except where otherwise indicated. Flies used in behavioral assays were collected by gentle aspiration or under light CO_2_ anesthesia shortly after eclosion (within 6 h), and aged 4–6 days in all the experiment except that of RNAi experiment and Gal80^ts^ experiment. Males were reared in groups of 8–10 animals or individually [18], [19], while females were kept in groups of 10 animals.

*Chaining Assay.* Eight males reared in groups were introduced into a 3.5 cm plastic dish by gentle aspiration or after immobilization by anesthetics. The courtship behavior between these males was video recorded and analyzed manually. The chaining behavior was defined as the display of courtship behavior among at least three males, with formation of a chain. The chaining index (ChI) is the percentage of time during which chaining behavior was observed [20]. For the antenna ablation experiment, the antenna (antennae) of males was (were) carefully removed by using fine forceps under light CO_2_ anesthesia in the first day after eclosion. Males were left to recover for 4 days before the chaining assay.

*Courtship Pairing Assay.* One individually reared tester male and one target female were sequentially introduced into a courtship chamber (diameter, 1 cm; height, 0.3 cm) by gentle aspiration, and the courtship behavior between the pair was video recorded and analyzed manually. The courtship index (CI) is the percentage of time in a given observation period during which the tester exhibits courtship behavior towards the target, which includes chasing, wing vibration, tapping, and attempted copulation [20].

*Courtship Preference Assay.* One individually reared tester male was introduced into a courtship chamber by gentle aspiration or by light CO_2_ anesthesia, and was left alone for 5 min. Two decapitated target flies, i.e., a *WT* male and a *WT* female, were then presented to the tester. The CI values towards the female and the male during a 10 min period were simultaneously measured and were subsequently compared [21].

*Olfactory Sensitivity.* Olfactory sensitivity was measured using a T-maze test [22], [23]. Briefly, flies were collected shortly after eclosion without anesthesia and were reared in groups until they were 2–3 days old. After being food deprived and kept in darkness for 0.5 h, ∼100 flies were introduced into the elevator of the T-maze and were left to choose between the two tubes, one filled with air and the other with MCH (Fluka, USA), for 2 min. The flies in each tube were then counted and the PI was calculated. All flies were reared on food without propionic acid and yeast, and the experiments were performed at 25±1°C with 75–90% relative humility in exclusive darkness with a red LED as a light source.

### RT–PCR

Adult males aged 2–3 days were lightly anesthetized by CO_2_ and dissected in cold (0°C) extracellular solution (ECS) containing the following: 103 mM NaCl, 3 mM KCl, 5 mM N-tris(hydroxymethyl) methyl-2-aminoethane-sulfonic acid, 10 mM trehalose, 10 mM glucose, 26 mM NaHCO_3_, 1 mM NaH_2_PO_4_, 1.5 mM CaCl_2_, and 4 mM MgCl_2_ (osmolarity was adjusted to 270–275 mOsm). About fifty proboscises and brains were separately collected in TRIzol reagent (Invitrogen, USA), and RNA was extracted according to the manufacturer's instructions. cDNAs from proboscises and brains were synthesized using standard techniques. The primers used to detect the presence of the *painless* cDNA were: 5′–GGAAACCTGGCGGCTCTCC–3′ and 5′– CAGCGGTGCCCTGGCCGA–3′.

### Immunostaining and Confocal Microscopy of Adult Brains

Two rabbit antisera against Painless were obtained from Novus Biologicals (NB100-98736 and NB100-98737) and were used at a dilution of 1∶200. Other antibodies (and dilutions) were as follows: rabbit anti-GFP (Invitrogen, 1∶1,000), mouse nc82 (Developmental Studies Hybridoma Bank, 1∶50), biotinylated goat antirabbit (GAR) IgG (Vector Labs, 1∶800), rhodamine avidin D (Vector Labs, 1∶500), and Alexa-fluor-conjugated secondary antibodies (Invitrogen, 1∶200). Flies aged 3–5 days were lightly anesthetized by CO_2_, fixed in PBS with 4% paraformaldehyde on ice for 1 h, washed and dissected in PBS with 0.2% Triton X-100 (PBST), and blocked for 1 h at 25°C in PBST containing 10% heat inactivated normal goat serum. For experiments using anti-Painless antibodies, brains were first incubated with anti-Painless antiserum for 8 h at 4°C. After washing in PBST (4×15 min), brains were incubated with biotinylated GAR IgG and nc82 for 8 h at 4°C. The brains were then washed in PBST (4×15 min) and incubated with rhodamine avidin D and Alexa-fluor-633-conjugated goat anti-mouse (GAM) IgG. For other stainings, brains were first incubated with anti-GFP and nc82 antibodies for 8 h at 4°C. After washing in PBST (4×15 min), brains were incubated with Alexa-fluor-488-conjugated GAR and Alexa-fluor-546-conjugated GAM IgGs. After final washes in PBST (3×5 min), brains were mounted in VECTASHIELD (Vector Labs). Confocal images were captured using a Carl Zeiss LSM510 microscope equipped with a Plan-Apochromat 20× objective or a 63× oil-immersion objective.

### Blocking Neurotransmission using shibire*^ts^*

For the experiments that required the use of *UAS-shibire^ts^*, flies were kept at 19°C until the experiment. To shift the environment temperature, flies were housed in 3.5 cm dishes made of aluminum and placed on a custom-built heating plate that can shift between 19 and 30°C within 1 min.

### Data analysis

Student's *t-*test was used to compare the intensity of courtship behaviour, after testing the normality of the data distribution with the Kolmogorov-Smirnov test. Otherwise, the Kruskal-Wallis test was used.

## Results

### Painless Is Expressed in Adult Fly Brain

**Figure 1:**
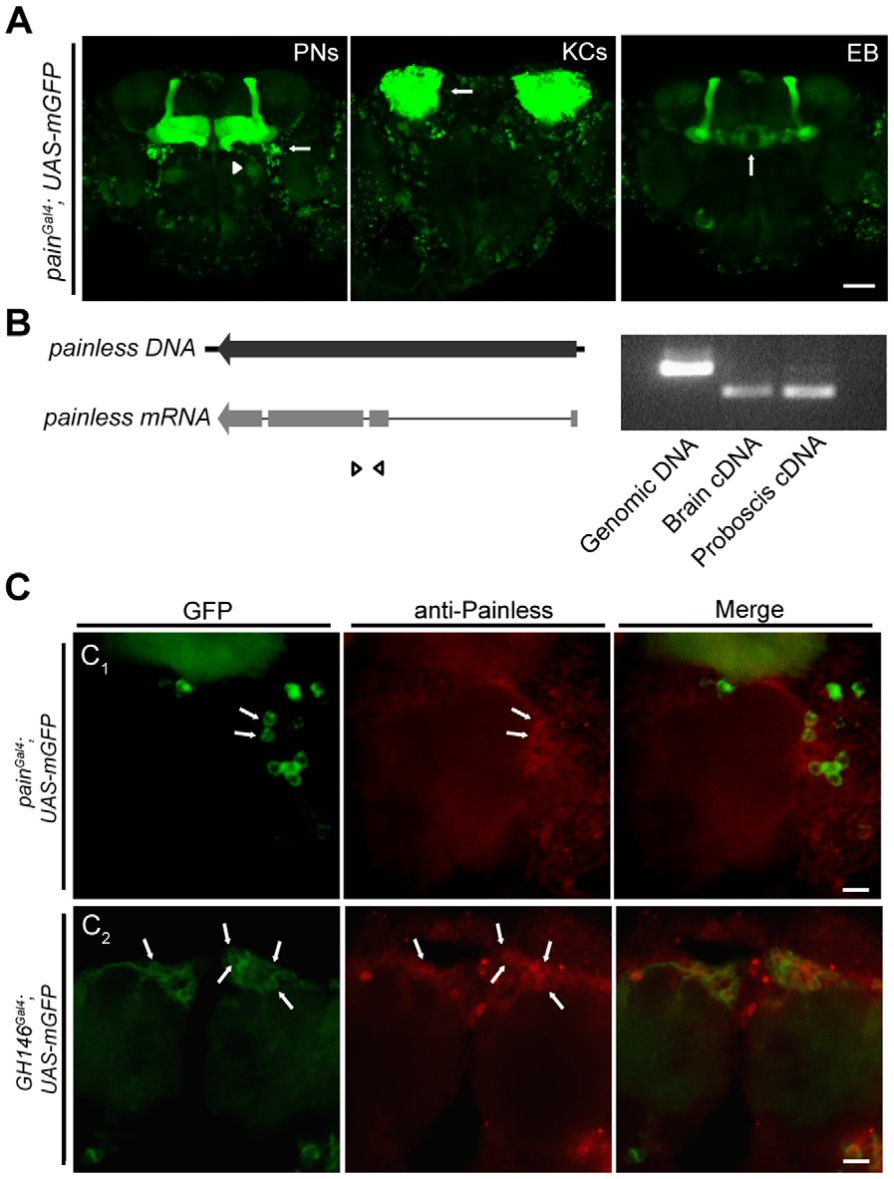
Expression of *painless* in the central nervous system. (*A*) Confocal images of the brains of *pain^Gal4^*; *UAS-mCD8-GFP* flies. Cells in green were *pain^Gal4^-positive*. White arrows indicate the projection neurons (PNs), the Kenyon cells (KCs), and the ellipsoid body (EB), respectively. White arrowhead in (*A*) shows one glomerulus innervated by neurites of pain^Gal4^-positive PNs (Scale bar, 50 µm.) (*B*) Left, schematic structure of the *painless* gene locus and mRNA. Small triangles represent the PCR primers used. Right, RT–PCR products of mRNAs from brains and proboscis, with PCR product from genomic DNA as a control. (*C*) Confocal images of fly brains stained with the antibody against Painless. The genotype of each brain is indicated. White arrows show the PNs expressing both Painless and GFP. White arrowhead in (*C_1_*) indicates the glomerulus formed by the neurites of GFP–positive PNs. (Scale bar, 10 µm.)

The Gal4 expression pattern in the Gal4 enhancer-trap line *pain^Gal4^*, which contains Gal4 inserted within the *painless* transcriptional unit [6], revealed that this Gal4 is broadly expressed in the adult brain [10]. Using the same Gal4 line, we found that *painless* is expressed in several clusters of PNs of the ALs, in Kenyon cells (KCs) of mushroom bodies (MBs), and in the neurons that form the ellipsoid body (Figure 1*A*). To confirm the endogenous expression of *painless* in the adult fly brain, we performed RT–PCR to examine the presence of the *painless* mRNA. As shown in Figure 1*B*, *painless* mRNA was detected in dissected brains of *WT Canton-S* (*CS*) adult flies, indicating the endogenous expression of the *painless* gene in the adult brain. We further confirmed the endogenous expression of *painless* by immunostaining. The specificity of the Painless antibody was first validated by specific staining of the Painless-myc fusion protein in cell lines overexpressing the protein, and detection of overexpression Painless protein in adult brains (Figure S1). We next examined the expression pattern of endogenous *painless* in adult brains using GFP expressed in PNs of the ALs (*pain^Gal4^*; *UAS-mGFP* and *GH146^Gal4^*; *UAS-mGFP*). As shown in Figures 1*C* _1_ and 1*C* _2_, Painless signal was detected in the circumference of the ALs, where the somata of olfactory PNs and LNs are located, and co-staining experiments showed that Painless was expressed in certain PNs. Also, we observed that some glomeruli, which receive the dendrites of PNs, were labeled by the antibody (Figure S1), suggesting a function of Painless in the dendrites. Although strong Painless signal was not detected in other brain regions, such as MBs, we could not exclude the possibility that Painless was also expressed in KCs and other neurons at relatively low levels.

### Olfaction Is Affected in *painless* Mutant Flies

As expression of endogenous Painless was detected in the PNs, we speculated that *painless* may function in the olfactory pathway, and contribute to the olfactory behavior. In order to test this possibility, we examined the olfactory sensitivity of *painless* mutant flies using the T-maze test [22], [23]. As shown in Figure S2, when provided with the odorant 4-methyl-cyclohexanol (MCH), *WT* flies preferred the tube perfused with air to that perfused with MCH. And as the concentration of MCH increased, the MCH avoidance behavior became stronger. Interestingly, this MCH avoidance behavior was significantly weakened in *painless* mutant flies (*pain^Gal4^* and *pain^1^*), suggesting that olfactory sensitivity of these flies was affected. When MCH was provided at lower concentrations (5.0×10^−6^ and 5×10^−7^, v/v dilution), *painless* mutant flies still displayed a preference behavior, suggesting an alteration in the responses towards this odor. To further test whether this olfactory defect was due to mutation of *painless*, we examined the avoidance behavior of *painless* mutant flies overexpressing Painless in *pain^Gal4^* positive neurons. We found that the avoidance behavior could be partially restored to a level resembling that of *WT* flies at high concentrations of MCH, but not at low concentrations (Figure S2). These results indicate that *painless* is involved in olfactory processing in flies.

### Male–male Courtship Behavior in *pain^Gal4^* Flies

**Figure 2:**
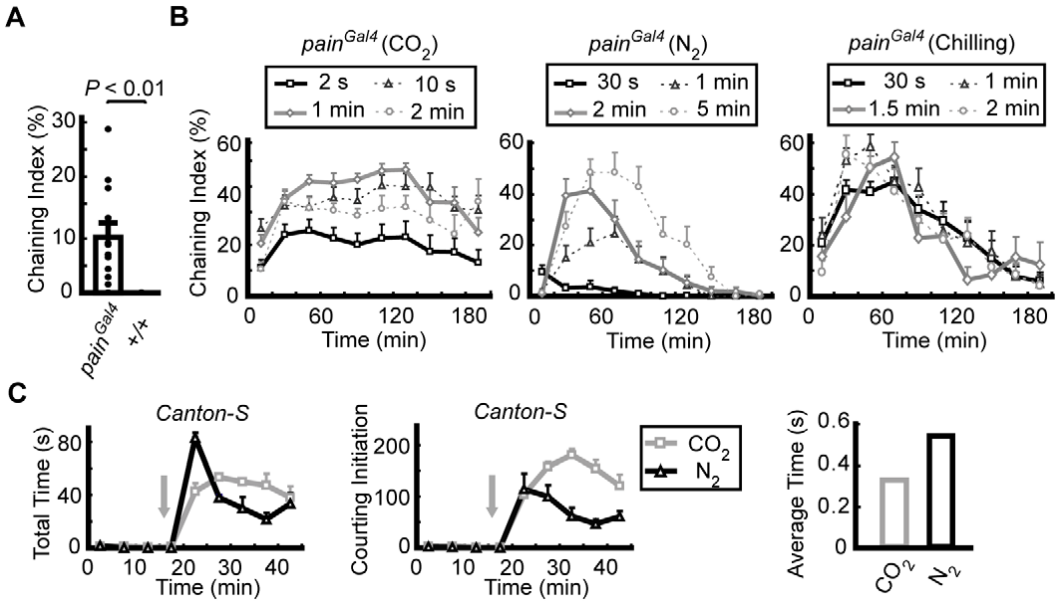
*painless* mutant males exhibited a male–male courtship behavior. (*A*) The average Chaining index (ChI) of 8–10 males that displayed spontaneous chaining behavior during a 10 min period in the morning (ZT0.5–ZT1.5). For both *WT* and *pain^Gal4^*males, more than six groups of males were analyzed. *P*<0.01 vs. the *WT* (Kruskal-Wallis test). (*B*) Average ChI of males recovered from the indicated anesthesia (top). Three different anesthetics were used in four different doses (indicated in the boxes above the traces). For each trace, 12 groups of males were observed every 5 min with a 15 min interval. (*C*) Average time spent by eight *WT* males on courting (left) and average time of courtship initiation (middle), before and after the indicated anesthesia treatments (grey arrows). Average time spent by the *WT* males on each courtship bout after anesthesia treatment (right). Values shown are means ± SEM.

In addition to the olfactory defects, we observed that male *pain^Gal4^* flies, in which the expression of *painless* was interfered by the Gal4 insertion, spontaneously displayed a male–male courtship behavior in the morning (zeitgeber time: ZT0.5–ZT3) (Figure 2*A*). We measured the chaining index (ChI) of the males, i.e., the percentage of time spent courting one another and forming chains. We found that the ChI of male *pain^Gal4^* flies was ∼10%, significantly higher than that of *WT* males (∼0%). We fortuitously observed that, after recovery from a mild anesthesia (brief treatments with CO_2_, nitrogen, or −20°C chilling), the intensity of this male–male courtship behavior was greatly enhanced over a long period (up to ∼3 h after CO_2_ treatment, Figure 2*B*). Interestingly, this anesthesia-induced enhancement of the male–male courtship behavior was not specific to *painless* mutant flies, as a brief anesthesia with either CO_2_ or nitrogen also induced a partial male–male courtship behavior lasting a very short period (0.3–0.5 s) in *WT* flies, i.e., an increase in the occurrence of courtship initiation (Figure 2*C*), which included the orientation, chasing, and wing vibration steps, but not followed by other courtship steps as in the *painless* mutant males. These observations suggest that male-male courtship behavior could be triggered among flies after recovery from a treatment of mild anesthesia, and *WT* males are able to inhibit this abnormal courtship behavior whereas *painless* mutant males cannot. Thus, in the following experiments, we treated male flies with brief CO_2_ anesthesia (10 s or 1 min) before the chaining assay to examine their ability to inhibit male-male courtship behavior.

### Male–male Courtship Behavior Is Caused by *painless* Mutation

**Figure 3:**
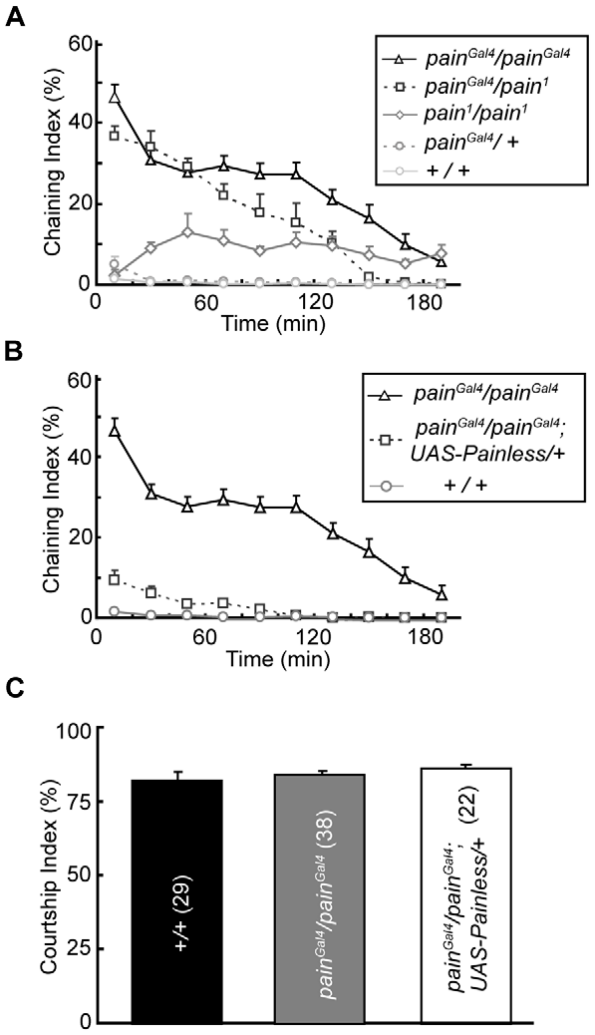
Mutation of *painless* caused a male–male courtship behavior. (*A*) and (*B*) Average ChI of males of indicated genotypes during the 3 h session. For each trace, more than ten groups of males was analyzed. Note that the experiments in (*A*) and (*B*) were performed simultaneously, and some data (ChI of *WT* and *pain^Gal4^*) was used in both panels. (*C*) Average courtship index of males of indicated genotypes towards *WT* virgin females. The number of males examined is shown in parenthesis. Histograms represent the mean ± SEM.

To confirm that the male–male courtship behavior of *pain^Gal4^* flies was caused by mutation of the *painless* gene, we examined the behavior of other mutant alleles of *painless*. We found that *painless^1^* (*pain^1^*) males exhibited long-lasting male–male courtship behavior after recovery from mild CO_2_ anesthesia, with a ChI lower than that of *pain^Gal4^* males, but significantly higher than that of *WT* males (Figure 3*A*). In addition, we performed complementation experiments and found strong male–male courtship behavior in *pain^Gal4^/pain^1^* double-heterozygous flies, with an average ChI as high as that observed in *pain^Gal4^* homozygous males. In contrast, *pain^Gal4^/+* and *pain^1^/+* heterozygous male flies did not display obvious male-male courtship behavior. In addition to *pain^Gal4^/pain^1^* males, we also examined the behavior of *pain^1^/pain^3^*, *pain^Gal4^/pain^3^*, and *pain^Gal4^/pain^4^* heterozygous males and found that the intensity of their male–male courtship behavior was significantly higher than that of *WT* animals (Figure S3), but shorter and weaker as compared with *pain^Gal4^/pain^1^* males.

Further rescue experiments showed that overexpression of Painless suppressed the male–male courtship behavior of *pain^Gal4^; UAS-Painless/+* flies (Figure 3*B*). Moreover, the courting ability of these Painless-overexpressing males towards *WT* virgin females was similar to that of *WT* males (Figure 3*C*). These results demonstrate that the abnormal courtship behavior found in *pain^Gal4^* mutant males was due to the *painless* mutation.

### Courtship Preference of *pain^Gal4^* Males towards Females

**Figure 4:**
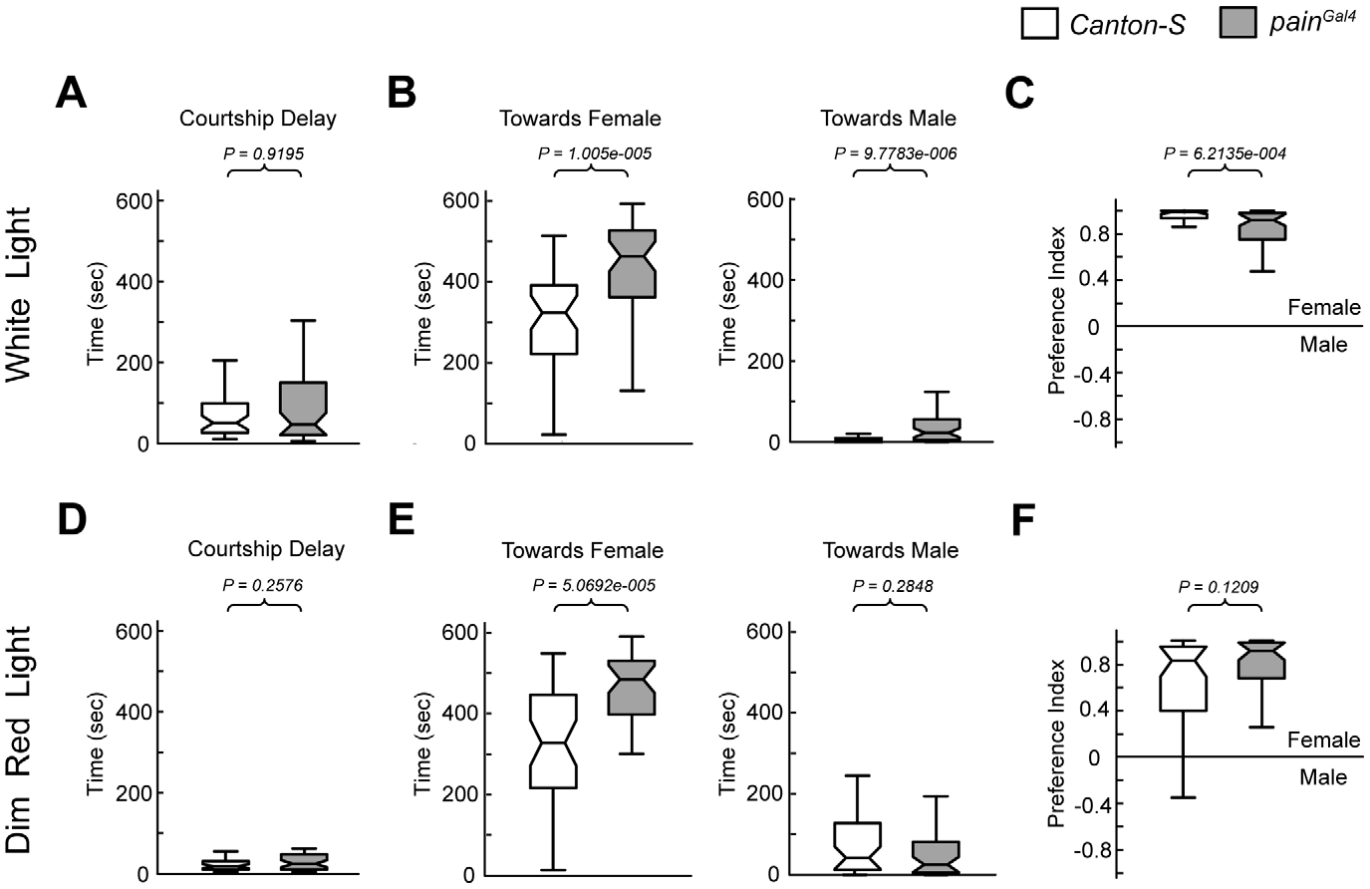
*painless* mutant males were capable of distinguishing female from male flies. (*A*) Box plots of courtship delay, which represents the latency before males initiated courtship behavior. (*B*) Total time spent by each individual male on courting decapitated females (left) and decapitated males (right). (*C*) Average PI of males of indicated genotypes towards females vs. males. Error bars represent SEM. (*D–F*) are similar as (*A–C*) except that these experiments were performed under dim red light instead of under white light as in (*A–C*). *P* values were determined by the Kruskal-Wallis test, and 43–48 males were examined in each group.

As the olfactory sensitivity to MCH was decreased in *painless* mutant flies, we next assessed whether the sensitivity of these flies to specific pheromones was also altered. If so, this could affect the ability of males to distinguish females from males and, thus be a cause for abnormal courtship behavior. We performed a courtship preference assay by measuring the preference index (PI) of *painless* mutant males towards decapitated male and female flies. As shown in Figure 4*A*, when placed with two decapitated *WT* flies of opposite genders, *pain^Gal4^* males and *WT* males displayed a similar delay in courtship initiation, suggesting that *pain^Gal4^* males were capable of sensing the targets and of initiating courtship behavior. We then examined the time spent by the *pain^Gal4^* male and *WT* males courting the two decapitated targets and found that *pain^Gal4^* males were able to distinguish female from male flies, although the PI was slightly lower as compared with that of *WT* flies (Figure 4*B and 4C*). These results suggest that the male–male courtship behavior of *painless* mutant flies was unlikely to be caused exclusively by their inability to differentiate between female and male flies. In addition, we noticed that the total time spent by *pain^Gal4^* males on courting was significantly longer than that observed for *WT* males (Figure 4*B*).

We also performed the experiment under dim red light to remove effects of the visual cues. We found that under such condition, both *WT* males and *pain^Gal4^* mutant males showed a shorter courtship delay (Figure 4*D*). In addition, *WT* males spent more time in courting the male target (Figure 4*E*), suggesting that visual input is important for the *WT* males to distinguish males from females. In contrast, *pain^Gal4^* mutant males showed similar sex-discriminating ability as that under white light (Figure 4*B and 4F*). Thus, it is possible that *pain^Gal4^* males are incapable of sensing inhibitory visual cues, or *pain^Gal4^* males have additional deficiencies which mask the vision-deprivation effect.

### Painless Expression in PNs Inhibits Male–male Courtship Behavior

**Figure 5:**
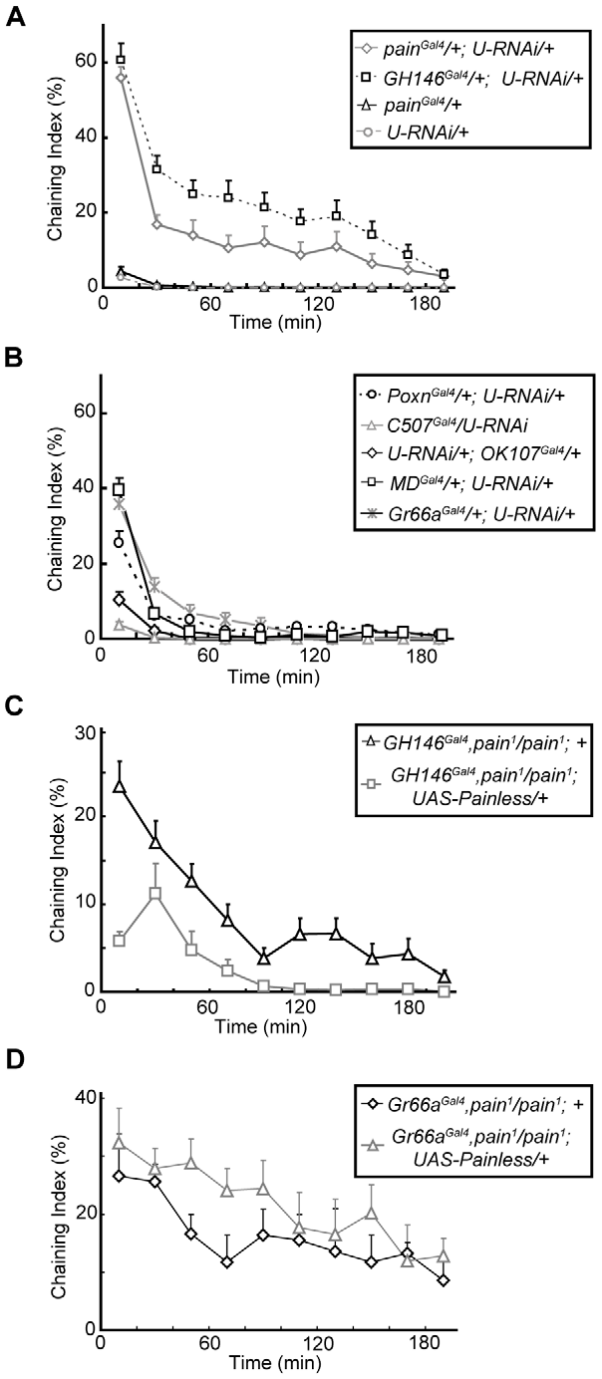
The expression of Painless in PNs inhibited the male–male courtship behavior. (*A* and *B*) Male–male courtship behavior caused by down-regulation of *painless* expression in different brain regions. (*C* and *D*) Overexpression of *painless* in *GH146^Gal4^*-positive neurons, but not in *Gr66a^Gal4^*-positive neurons, suppressed the male–male courtship behavior in *painless* mutant males. Average ChI of males of indicated genotypes during the 3 h observing session are shown. For each trace, more than eight groups of males were analyzed. Error bars represent SEM.

The above-described results showed that disruption of the *painless* gene led to decreased olfactory sensitivity and male–male courtship behavior. However, the brain regions in which Painless expression was required for the prevention of this aberrant behavior are unknown. The expression pattern of *painless* suggested a role for *painless* in olfactory processing. Therefore, we manipulated Painless expression in the olfactory PNs of the ALs. We first used two RNAi transgenic lines targeting *painless* [24] (*UAS-pain-RNAi-1* and *UAS-pain-RNAi-2*) to downregulate Painless expression globally under the control of *pain^Gal4^*. As shown in Figures 5*A* and Figure S4, global downregulation of Painless expression in *pain^Gal4^*-positive neurons using either RNAi line induced male–male courtship behavior, while *pain^Gal4^/+* heterozygous flies and the two *UAS-pain-RNAi*-alone flies did not exhibit this behavior. These results suggest that these two RNAi lines effectively downregulated Painless expression and induced the male–male courtship behavior.

We next used different Gal4 lines to knockdown Painless expression preferentially in selective subsets of neurons in an attempt to localize the site of action of Painless during the regulation of courtship behavior. We found that preferential knockdown of Painless expression in about two thirds of the olfactory PNs [25] using a *GH146^Gal4^*-driven RNAi caused severe male–male courtship behavior (Figure 5*A*). Downregulation of Painless expression in certain gustatory receptor neurons (GRNs) using RNAi driven by *Gr66a^Gal4^*, *Poxn^Gal4^*, or *MD^Gal4^* resulted in weak male–male courtship behavior (Figure 5*B*). As KCs in MBs and the neurons in the central complex were *pain^Gal4^*-positive, we further downregulated Painless expression in these two clusters of neurons by driving RNAi expression under the control of *OK107^Gal4^* and *C507^Gal4^*, respectively. No significant male–male courtship behavior was observed in these two fly genotypes (Figure 5*B*).

In parallel, we conducted rescue experiments to examine whether preferential overexpression of Painless in PNs or GRNs prevented the male–male courtship behavior caused by the *painless* mutation. As shown in Figures 5*C and 5D*, the intensity and duration of the male–male courtship behavior in *pain^1^* flies were significantly reduced following preferential expression of Painless in the *GH146^Gal4^* labeled PNs. However, overexpression of Painless in *Gr66a^Gal4^*-positive neurons did not prevent male–male courtship behavior. These results of the cell-type-specific knockdown and rescue experiments suggest that the expression of Painless in PNs is necessary and sufficient for the suppression of male–male courtship behavior.

### Male–male Courtship Behavior in *pain^Gal4^* Flies Is Not Caused by Developmental Defects

**Figure 6:**
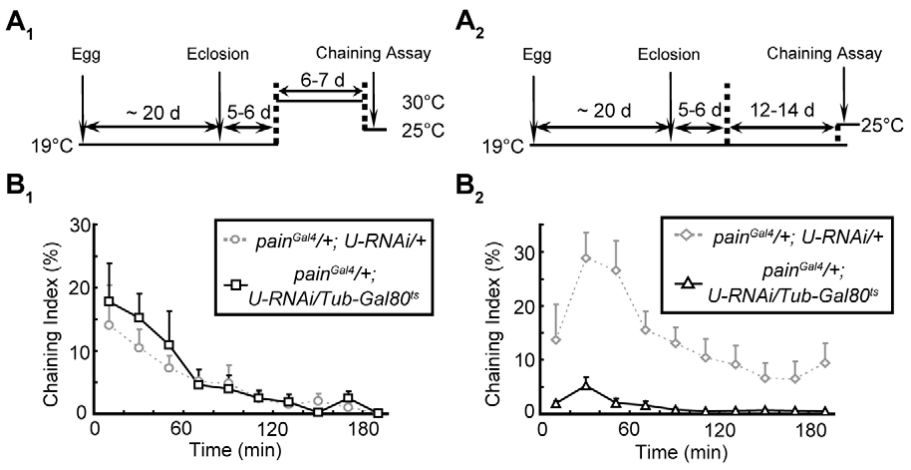
Downregulation of the expression levels of *painless* exclusively in adulthood resulted in male–male courtship behavior. (*A_1_* and *A_2_*) Schematic representations of the experimental design used to manipulate the expression levels of *painless*. Flies were raised and maintained at different temperatures, as indicated. Average ChI of males of indicated genotypes reared according to the strategies shown in *A_1_* (*B_1_*) or *A_2_* (*B_2_*). Error bars represent SEM, and n>8 in each trace.

To determine whether the male–male courtship behavior is caused by developmental defects associated with *painless* mutation, we used the temporal and regional gene-expression targeting (TARGET) system to switch on RNAi expression in adult flies in a time-specific manner [26]. We used the temperature-sensitive Gal80 protein (Gal80^ts^) to manipulate the activity of Gal4. The effectiveness of Gal80^ts^ was shown by the following experiments. The maintenance of flies carrying a copy of Gal80^ts^ at a permissive temperature (19°C) led to negligible detection of GFP signal in the brain of *painGal4;UAS-mGFP/TubP-Gal80^ts^* animals. However, the transfer of the flies to the restrictive temperature (30°C) for 5–6 additional days restored GFP expression (Figure S5). We then used the TARGET system to switch on RNAi expression in adult flies. As shown schematically in Figure 6*A*, we inhibited the expression of *UAS-pain-RNAi* during early development by maintaining the flies at 19°C until 5–6 days after eclosion to ensure their proper development, and then relieved the inhibiting activity of Gal80 to restore the RNAi expression by elevating the temperature to 30°C for additional 5–6 days. Male–male courtship behavior was observed in these flies, with intensity and duration similar to those observed for the *RNAi* flies not expressing Gal80^ts^ (Figure 6*B*). This result indicates that downregulation of *painless* in adult flies was sufficient to induce male–male courtship behavior.

## Discussion

To study the physiological role of Painless in the central nervous system of adult fruit flies, we examined the behavior of *painless* mutant flies. We found that the *painless* mutation significantly increased male–male courtship behavior (Figures 2 and 3). By altering the expression level of Painless in a subpopulation of PNs in ALs, we were able to induce this male-male courtship behavior, suggesting that Painless expression in the olfactory system was necessary for inhibition of this abnormal courtship behavior (Figure 5). Furthermore, temporally specific Painless knockdown in the adult fly was sufficient to induce the male-male courtship phenotype (Figure 6). Moreover, mutation of the *painless* gene reduced olfactory sensitivity (Figure S2). Together, our results suggest that the expression of *Drosophila* Painless in a subset of PNs is essential for normal male courtship behavior. Because PNs are the principal relay neurons of the *Drosophila* olfactory system, Painless may play an important role in gating olfactory information; thus, mutation of this protein may lead to deficits in odor/pheromone perception and aberrant courtship behaviors.

### Effects of Manipulating Painless Expression

In this study, the behaviors of several *painless* mutants were examined, and the expression level and patterns of *painless* were manipulated by using *UAS-pain-RNAi* and *UAS-Painless* driven by different Gal4 strains. We found that *painless* mutant males exhibited male-male courtship behavior (Figure 2 and Figure S3), at various intensities depending on the genotype. The *painless* mutants we used (*pain^1^*, *pain^3^* and *pain^Gal4^*) have been reported to have P-element insertions immediate upstream of the first non-coding exon of *painless* gene, resulting in the expression of mutant Painless proteins [6]. Thus, it is possible that the male-male courtship behavior observed in *painless* mutants is due to loss of *WT* Painless protein function, or the expression of mutant Painless proteins, or both. Our results showing that overexpression of *WT* Painless in the mutant background significantly inhibited male-male courtship behavior (Figure 3*B* and Figure 5*C*), and that knockdown of *WT* Painless expression induced male-male courtship behavior (Figure 5 and Figure S4) indicate that the loss of *WT* Painelss function is likely the major cause of this abnormal courtship behavior. However, we cannot exclude the possibility that the mutant Painless proteins resulting from the P-element insertion also contributes to the male-male courtship behavior, as the intensity of this behavior differed between *painless* mutant alleles.

By preferentially down-regulating Painless expression in different subsets of neurons, we found that the expression of *painless* in PNs was essential for the inhibiting courtship behavior among males. However, the contribution of *painless* expression in other neurons cannot be excluded. As shown in Figure 5*C*, preferential overexpression of *painless* in PNs of *painless* mutants did not fully suppress the abnormal courtship behavior. In addition, downregulation of *painless* expression in neurons (*Gr66a^Gal4^*, *Poxn^Gal4^*, and *MD^Gal4^*) other than PNs resulted in a mild male–male courtship behavior (Figure 5*B*). As Painless is expressed in a subset of GRNs that overlaps with the Gr66a-expressing neurons [10], involved in the detection of bitter substances and certain cuticular pheromones [27]–[29], we speculate that *painless* may also contribute to the sensation of these non-volatile pheromones by the *Drosophila* gustatory system [30]–[32].

### Role of Painless in Gender Preference in flies

Male flies use multiple sensory modalities to discriminate female from males. When confronting with two targets of opposite genders, *WT* males spent almost all their time courting the female target, while *painless* mutant males also courted the male target (Figure 4). This observation suggests that *painless* mutant males have deficency in sensing the inhibitory cues on the male targets. Combined with results obtained from down- or up-regulating Painless expression in subsets of neurons (Figure 5), we surmise that *painless* expression in the olfactory system is involved in sensing cues. Meanwhile, the gender-preference assay revealed that *painless* mutants exhibited a longer courtship period towards both females and males as compared with *WT* flies (Figure 4), suggesting a general enhancement of courtship activity. This alteration is also suggested by the finding that *painless* mutant females exhibit enhanced sexual receptivity [33]. Thus, *painless* may play a role both in perception of inhibitory cues from males and in gating the intensity of the courtship behavior.

### Effects of Anesthesia on Courtship Behavior

It is known that anesthesia affects the courtship behavior of individual males towards females [34]. The copulation latency of *WT* flies becomes longer after recovery from CO_2_ or chilling anesthesia [34]. Consistently, we found that brief anesthesia led to a significant decrease in the intensity of the courtship behavior of individual males (both *WT* males and *painless* mutants) towards decapitated females (Figure 4). However, the courtship behavior of an individual male towards another decapitated male was unchanged, while this behavior among a group of males was markedly enhanced. One explanation for these differential effects of anesthesia on male–male and male–female courtship behaviors is that anesthesia could weaken the sensation of attractive or repulsive pheromonal cues from females or males, respectively.

Distinct parts of the nervous system have differential sensitivities to anesthetics [35]. We speculate that in males there are neural circuits for sensing cues from other males to inhibit the male-male courtship behavior, and these circuits are highly sensitive to anesthetics. In addition, there are neural components which are responsible for the exhibition of courtship behavior, which are less sensitive than the inhibitory circuits. In the presence of mild anesthetics, e.g., brief CO_2_, nitrogen, and chilling used in the present study, the inhibitory neural circuits may loss their function more easily than the neural circuits responsible for courting, causing the animals to exhibit male-male courting. As the dose of anesthetics increased, the neural circuits responsible for courting were also disabled, resulting in decreases and eventually abolishment of courtship behavior. (Figure 2*B*).

We also noticed that after recovery from an anesthesia of same dose, two *painless* mutants with the same P-element insertion (*pain^1^* and *pain^Gal4^*) exhibited male-male courtship behavior of distinct intensities and temporal patterns (Figure 3). This is probably due to differences between the natures of these two *painless* mutant alleles (e.g. motility, vision). Furthermore, this might be due to the different sensitivity to anesthesia in *pain^1^ and pain^Gal4^* mutants, as the recovery times from anesthesia in *pain^1^* was longer than that in *pain^Gal4^* males (Figure S6).

We also tested the idea whether anesthesia-sensitive neural circuits have some overlap with the *painless* circuits. By time-specific blockade of the neural transmission of *pain^Gal4^*-expressing neurons with a temperature-sensitive Shibire protein [36], a male–male courtship behavior could be triggered in the absence of anesthetics (Figure S7), suggesting that Painless might function in some courtship-inhibiting neural circuits which are also anesthesia-sensitive. Therefore, the enhanced courtship behavior observed among the *painless* mutant males after recovery from anesthesia should comprise integrated effects of both the impairment of *painless* function and anesthesia.

The mating behavior of *painless* mutant male flies towards other male flies may be caused by removal of the inhibition mechanism that prevents a male fly from pursuing other male flies. Removal of the inhibition mechanism in *painless* mutant flies is probably caused by their inability to sense the signal from other male flies correctly. Our results showing that the loss of function of the *painless* gene in PNs was essential for male–male courtship behavior, and that olfactory sensitivity was decreased in *painless* mutant flies, suggest that olfactory perception in *painless* mutant flies is impaired and may result in their inability to perceive inhibitory chemical cues (e.g. *cis*-Vaccenyl acetate) from other male flies effectively [30], [37], [38]. As one of the members of the TrpA family of ion channels, the expression of Painless in PNs may contribute to the electrophysiological properties of these neurons (e.g., the excitability and firing pattern of PNs). Thus, we speculate that *painless* may be involved in the coding of the olfactory/pheromone signals in PNs, which are important for the inhibition of male–male mating.

## Supporting Information

Figure S1 (**A**) **COS cells overexpressing Painless-myc fusion protein (left) were labeled by anti-Painless and anti-myc antibodies, while COS cells transfected with control plasmid showed no detectable signals (right) (Scale bar, 50 µm).** (B) Confocal images of different peripheral organs of male flies of *pain^Gal4^*; *UAS-mGFP*, with green signal indicating GFP. Note that GFP was not detected in the third segment of antennae and the maxillary palps. (C) Confocal images of fly brains stained with the antibody against Painless. White arrows show the PNs expressing both Painless and GFP. White arrowhead indicates the glomerulus formed by the neurites of GFP–positive PNs. Some GFP-negative but Painless-positive neurons could be observed, suggesting that *pain^Gal4^* might not label all Painless-expressing neurons. (Scale bar, 10 µm.)
(TIF)

Figure S2 **Olfactory sensitivity was affected by** ***painless*** **mutation.** The average preference indices (PI) to different concentrations of MCH were examined using a T-maze assay (A), and the olfactory sensitivity of flies of indicated genotypes was shown in (B). For each point, 13–35 groups of flies were examined. *, *P*<0.05, **, *P*<0.01 vs. the *wild-type* group (Kruskal-Wallis test).
(TIF)

Figure S3 **Male-male courtship behavior in three** ***painless*** **mutant flies.** Average ChI of males of indicated genotypes during the 3 h observation session. For each trace, more than eight groups of males were analyzed. Error bars mean SEM.
(TIF)

Figure S4 **Expression of RNAi targeting** ***painless*** **in pain^Gal4^-positive neurons resulted in the male-male courtship behavior.** The traces show the average ChI of males of indicated genotypes. Error bars represent SEM. For each trace, more than eight groups were analyzed.
(TIF)

Figure S5 **Effectiveness of Gal80^ts^ in suppressing the transcriptional activity of Gal4.** The confocal images of brains of indicated genotype were shown. After maintained the flies at the restrictive temperature (30°C) for 6–7 days, GFP signal could be detected in pain^Gal4^-positive neurons. In contrast, maintenance of the flies at the permissive temperature (19°C) could effectively suppress the expression of GFP.
(TIF)

Figure S6 **Recovery time of males of indicated genotypes from a 15 s CO_2_ anesthesia.** Histograms represent the means, and error bars are SEM. No significant difference was found between the *WT* males and *pain^1^* males, whereas *pain^Gal4^* males have a shorter recovery time. P values were analyzed by Student's t test. The numbers of males examined are shown in parenthesis.
(TIF)

Figure S7 **Blockade of the neurotransmission of the pain^Gal4^-positive neurons resulted in the male-male courtship behavior.** The temperature was firstly shifted from 19°C to 30°C, and after maintaining for a period, was shifted back to 19°C. The behavior between eight males of indicated genotypes at either 19°C or 30°C were analyzed. Histograms show the average ChI, and error bars mean SEM. For each genotype, more than eight groups were analyzed.
(TIF)
