# Supplementary figures and images for: Drosophila TRPA Channel Painless Inhibits Male–Male Courtship Behavior through Modulating Olfactory Sensation
Source: PLoS One. 2011 Nov 2;6(11):e25890. doi: 10.1371/journal.pone.0025890 (PMC3206795; doi:10.1371/journal.pone.0025890)

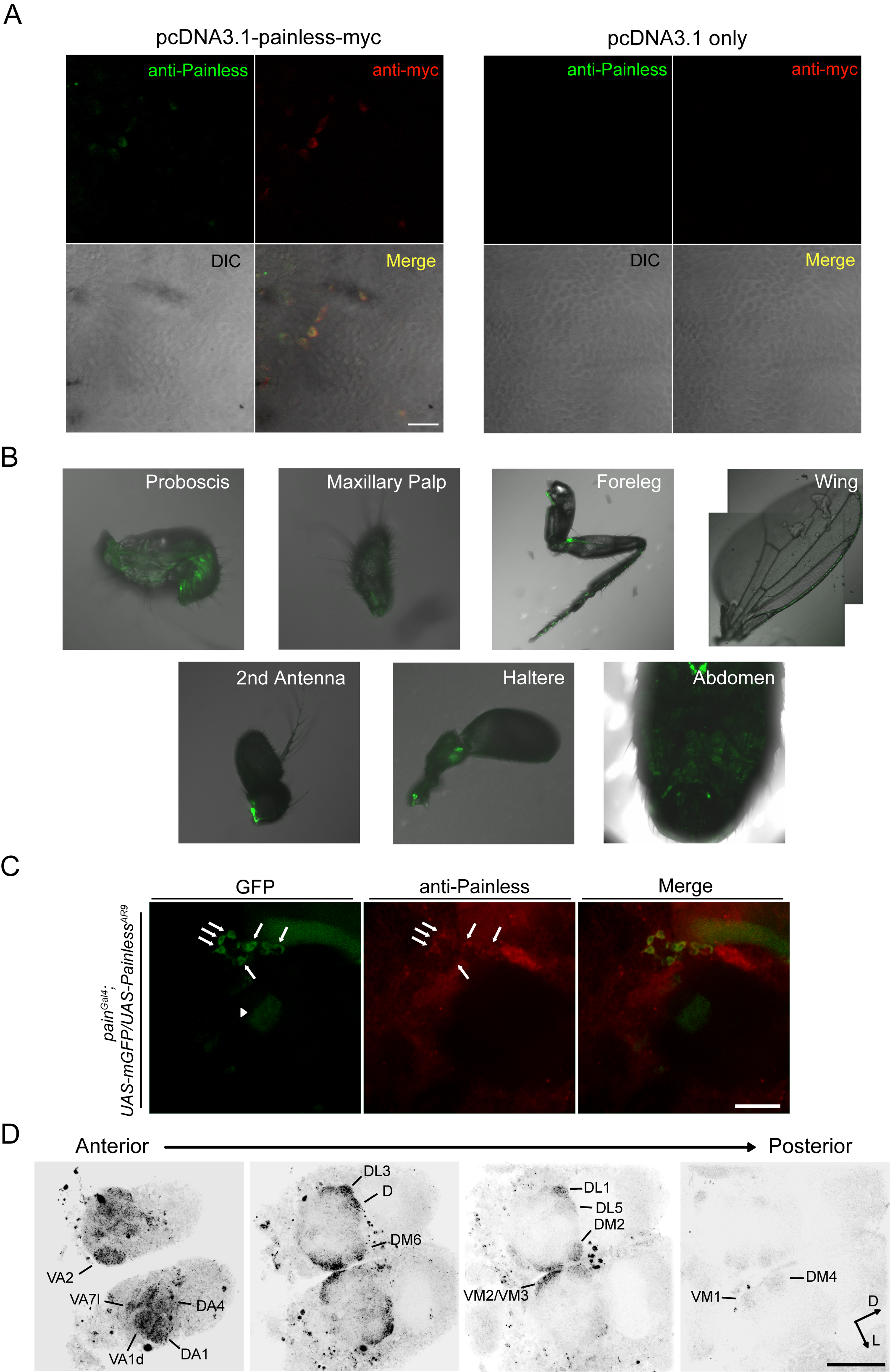

Supplement: Figure S1 — (A) COS cells overexpressing Painless-myc fusion protein (left) were labeled by anti-Painless and anti-myc antibodies, while COS cells transfected with control plasmid showed no detectable signals (right) (Scale bar, 50 µm). (B) Confocal images of different peripheral organs of male flies of painGal4; UAS-mGFP, with green signal indicating GFP. Note that GFP was not detected in the third segment of antennae and the maxillary palps. (C) Confocal images of fly brains stained with the antibody against Painless. White arrows show the PNs expressing both Painless and GFP. White arrowhead indicates the glomerulus formed by the neurites of GFP–positive PNs. Some GFP-negative but Painless-positive neurons could be observed, suggesting that painGal4 might not label all Painless-expressing neurons. (Scale bar, 10 µm.) (TIF) [file pone.0025890.s001.tif]

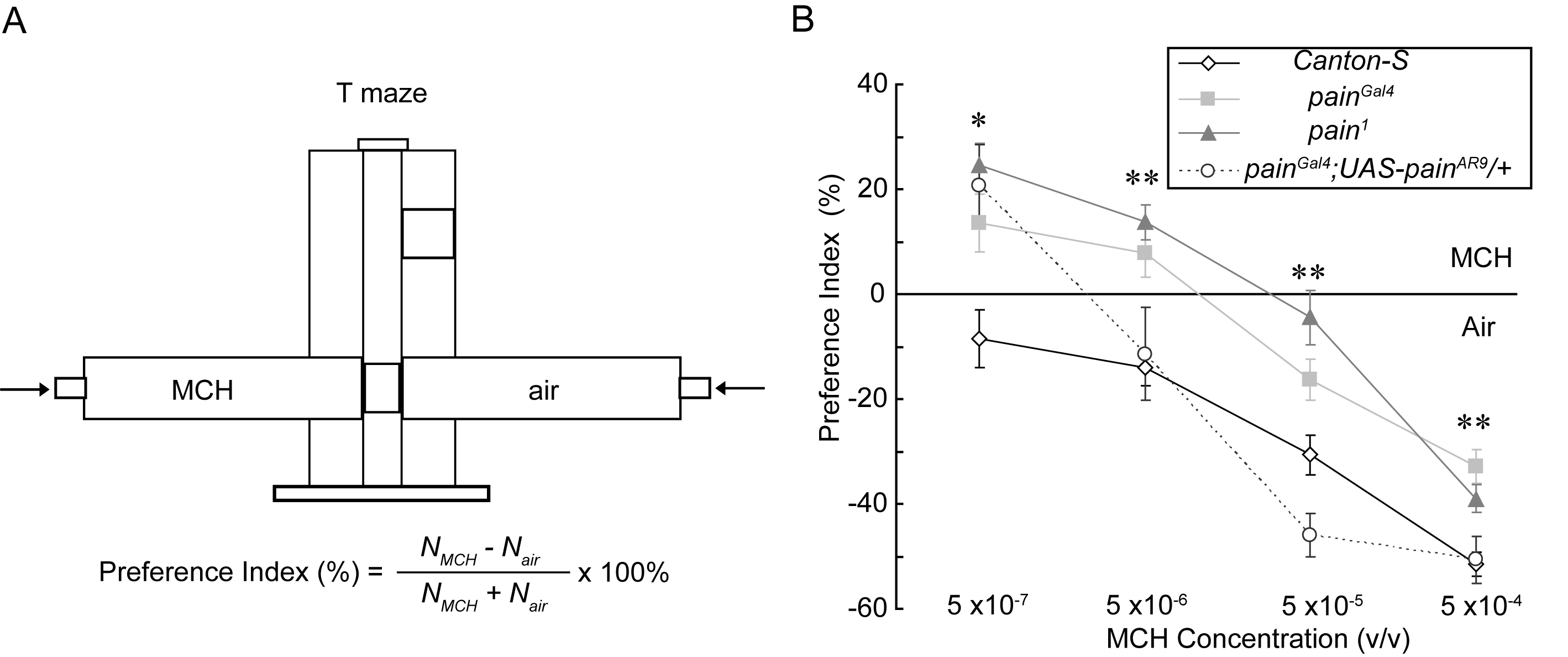

Supplement: Figure S2 — Olfactory sensitivity was affected by painless mutation. The average preference indices (PI) to different concentrations of MCH were examined using a T-maze assay (A), and the olfactory sensitivity of flies of indicated genotypes was shown in (B). For each point, 13–35 groups of flies were examined. *, P<0.05, **, P<0.01 vs. the wild-type group (Kruskal-Wallis test). (TIF) [file pone.0025890.s002.tif]

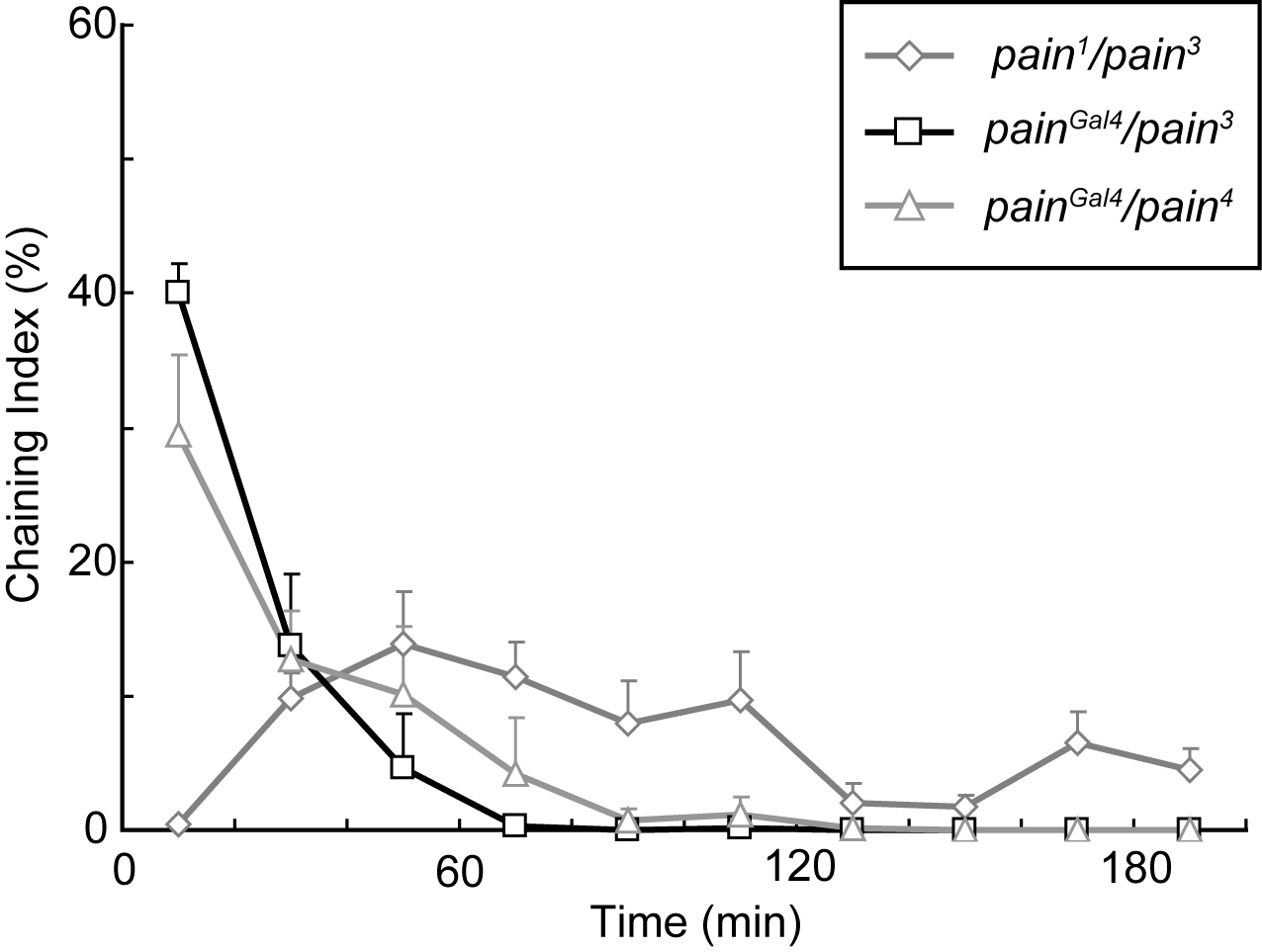

Supplement: Figure S3 — Male-male courtship behavior in three painless mutant flies. Average ChI of males of indicated genotypes during the 3 h observation session. For each trace, more than eight groups of males were analyzed. Error bars mean SEM. (TIF) [file pone.0025890.s003.tif]

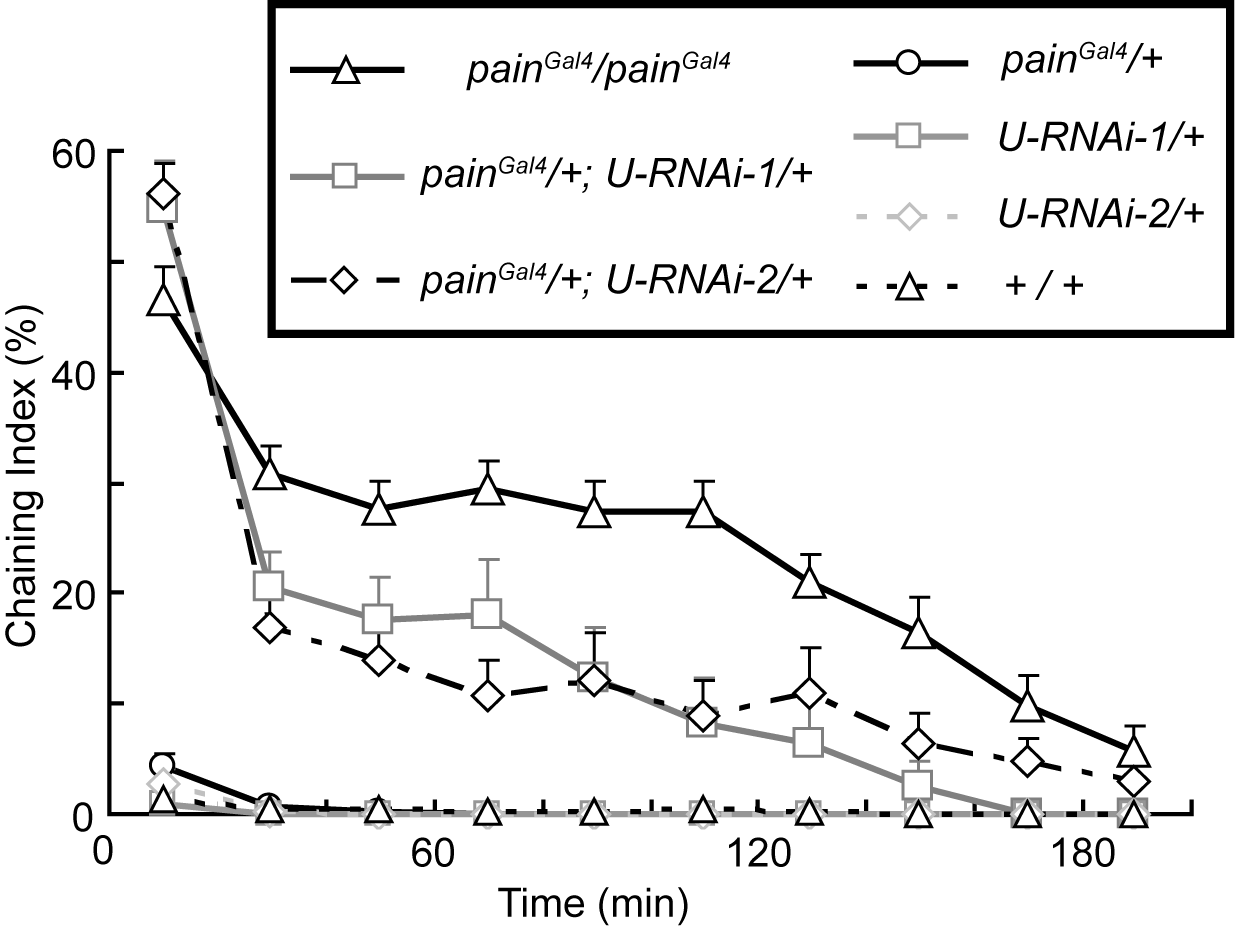

Supplement: Figure S4 — Expression of RNAi targeting painless in painGal4-positive neurons resulted in the male-male courtship behavior. The traces show the average ChI of males of indicated genotypes. Error bars represent SEM. For each trace, more than eight groups were analyzed. (TIF) [file pone.0025890.s004.tif]

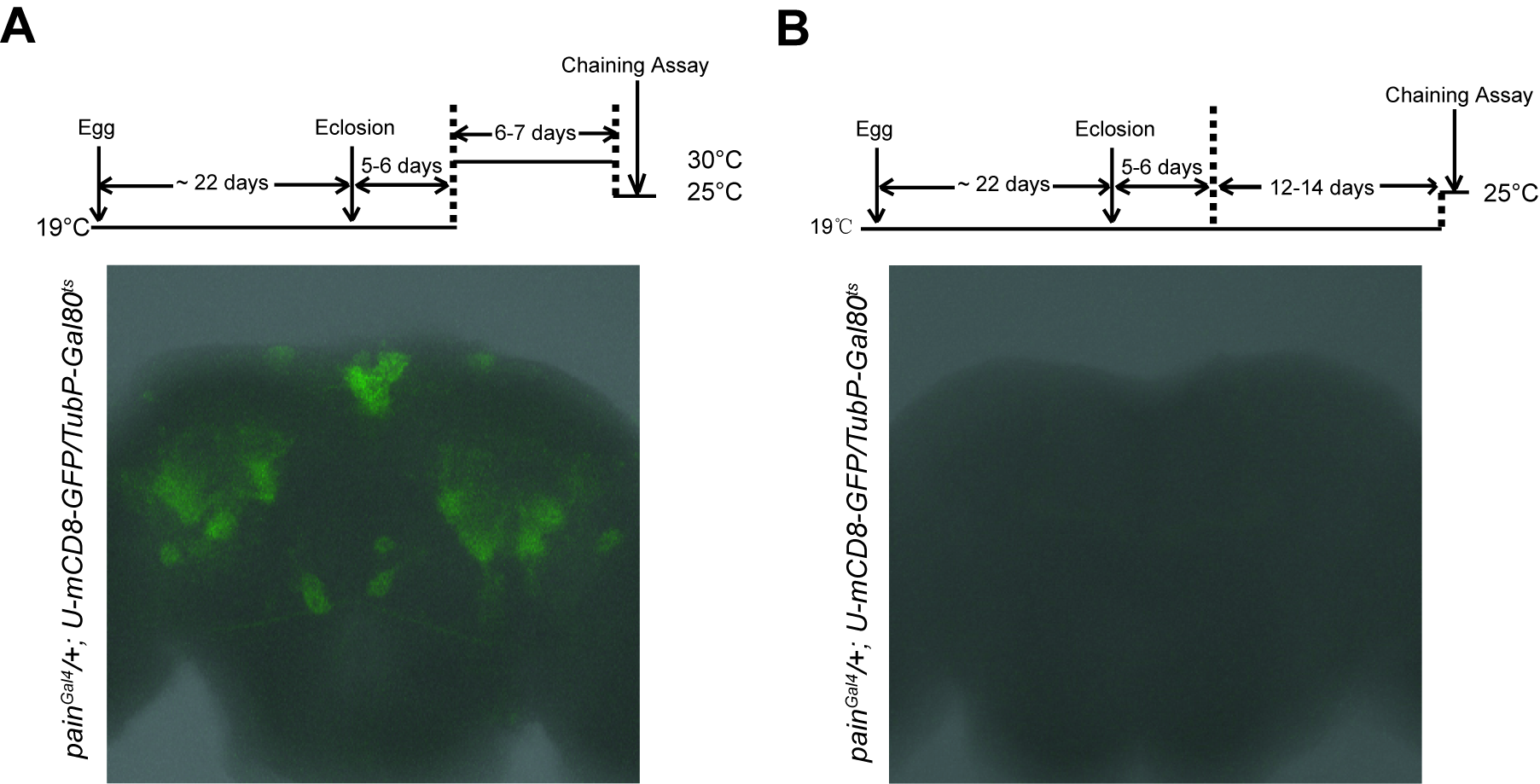

Supplement: Figure S5 — Effectiveness of Gal80ts in suppressing the transcriptional activity of Gal4. The confocal images of brains of indicated genotype were shown. After maintained the flies at the restrictive temperature (30°C) for 6–7 days, GFP signal could be detected in painGal4-positive neurons. In contrast, maintenance of the flies at the permissive temperature (19°C) could effectively suppress the expression of GFP. (TIF) [file pone.0025890.s005.tif]

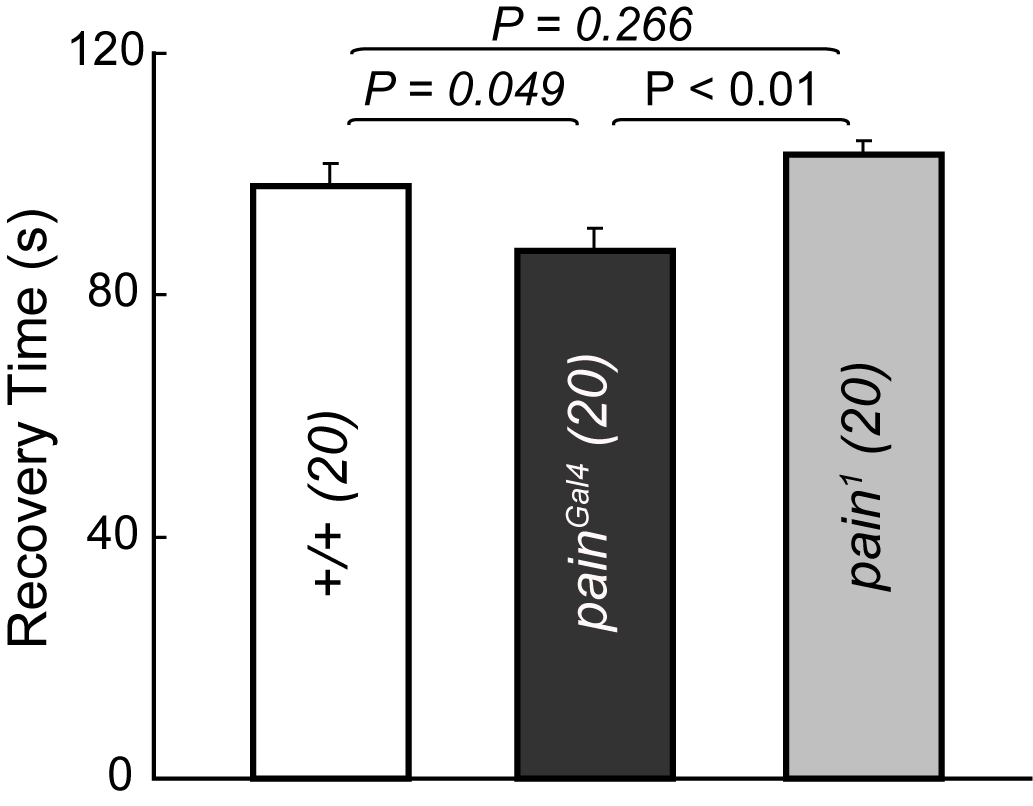

Supplement: Figure S6 — Recovery time of males of indicated genotypes from a 15 s CO2 anesthesia. Histograms represent the means, and error bars are SEM. No significant difference was found between the WT males and pain1 males, whereas painGal4 males have a shorter recovery time. P values were analyzed by Student's t test. The numbers of males examined are shown in parenthesis. (TIF) [file pone.0025890.s006.tif]

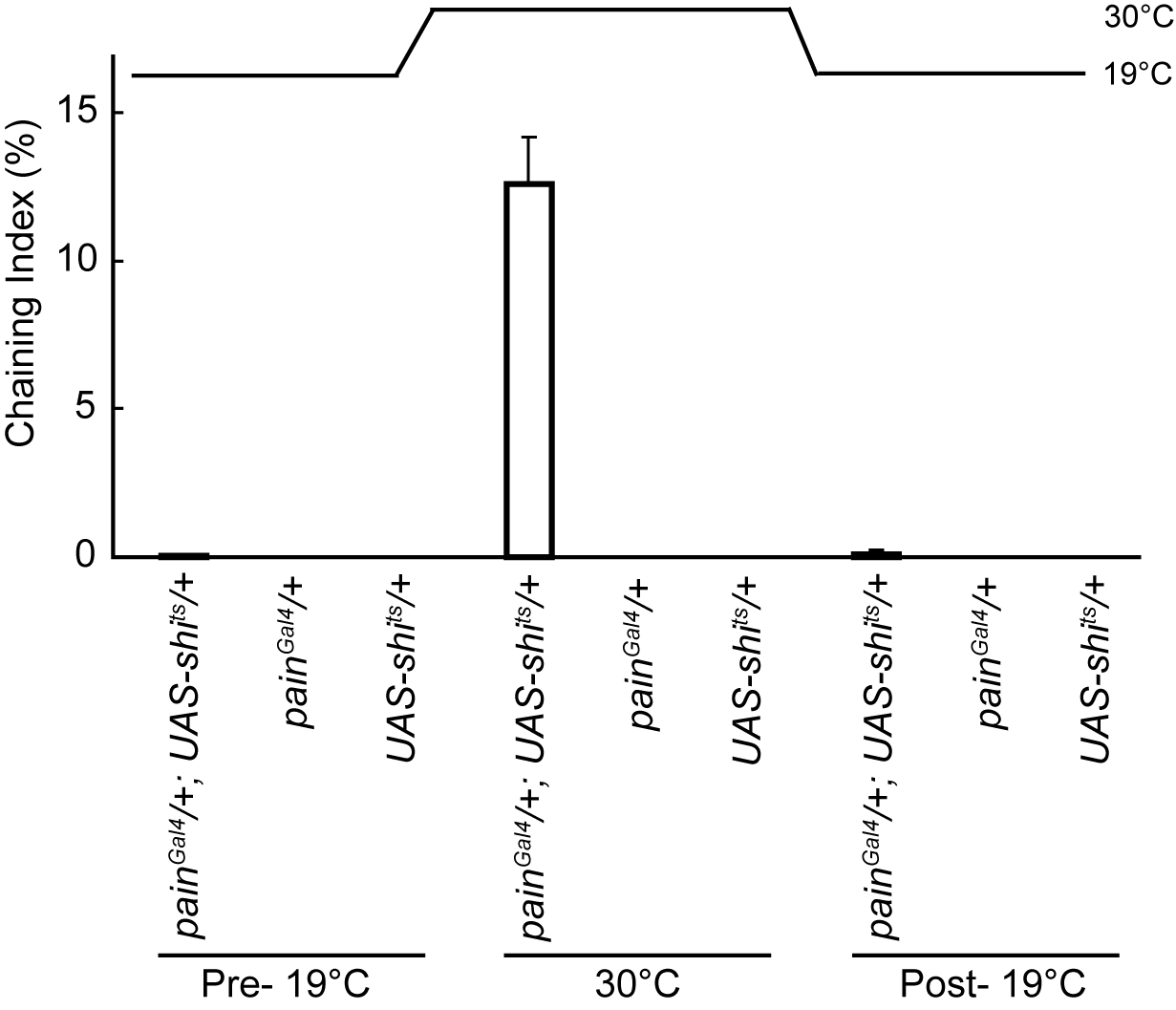

Supplement: Figure S7 — Blockade of the neurotransmission of the painGal4-positive neurons resulted in the male-male courtship behavior. The temperature was firstly shifted from 19°C to 30°C, and after maintaining for a period, was shifted back to 19°C. The behavior between eight males of indicated genotypes at either 19°C or 30°C were analyzed. Histograms show the average ChI, and error bars mean SEM. For each genotype, more than eight groups were analyzed. (TIF) [file pone.0025890.s007.tif]
